# A radiomics-based logistic regression model for the assessment of emphysema severity

**DOI:** 10.5578/tt.20239710

**Published:** 2023-09-22

**Authors:** M. Gulbay

**Keywords:** emphysema, machine learning, logistic regression

## Abstract

**ABSTRACT:**

A radiomics-based logistic regression model for the assessment of emphysema severity

**Introduction:**

The aim of this study is to develop a model that differentiates between the radiological patterns of severe and mild emphysema using radiomics parameters, as well as to examine the parameters included in the model.

**Materials and Methods:**

Over the last 12 months, a total of 354 patients were screened based on the presence of terms such as “Fleischner”, “CLE”, and “centriacinar” in their thoracic CT reports, culminating in a study population of 82 patients. The study population was divided into Group 1 (Fleischner mild and moderate; n= 45) and Group 2 (Fleischner confluent and advanced destructive; n= 37). Volumetric segmentation was performed, focusing on the upper lobe segments of both lungs. From these segmented volumes, radiomics parameters including shape, size, first-order, and second-order features were calculated. The best model parameters were selected based on the Bayesian Information Criterion and further optimized through grid search. The final model was tested using 1000 iterations of bootstrap resampling.

**Results:**

In the training set, performance metrics were calculated with a sensitivity of 0.862, specificity of 0.870, accuracy of 0.863, and AUC of 0.910. Correspondingly, in the test set, these values were sensitivity= 0.848; specificity= 0.865; accuracy= 0.857; and AUC= 0.907.

**Conclusion:**

The logistic regression model, composed of radiomics parameters and trained on a limited number of cases, effectively differentiated between mild and severe radiological patterns of emphysema using computed tomography images.

## Abstract (tr)

**ÖZ:**

Amfizem ciddiyetini değerlendiren radyomiks tabanlı lojistik regresyon modeli çalışması

**Giriş:**

Bu çalışmanın amacı, radyomiks parametreleri kullanarak ağır ve hafif amfizeme ait radyolojik paternleri ayırt eden bir model geliştirmek ve modele katılan parametreleri incelemektir

**Materyal ve Metod:**

Son 12 ay içerisinde, torasik BT raporlarında “Fleischner”, “CLE” ve “sentriasiner” terimleri geçen toplam 354 hasta incelendi ve 82 hastadan oluşan bir çalışma popülasyonu oluşturuldu. Çalışma popülasyonu Grup 1 (Fleischner hafif ve orta; n= 45) ve Grup 2 (Fleischner confluent ve advanced destructive; n= 37) olarak iki gruba ayrıldı. Vakalarda her iki akciğerin üst lob segmentlerine odaklanan volumetrik bir segmentasyon gerçekleştirildi. Bu segmente edilmiş hacimlerden şekil, boyut, birinci derece ve ikinci derece radyomiks parametreleri hesaplandı. En iyi model parametreleri Bayesyen Bilgi Kriteri temel alınarak seçildi ve Grid Search ile optimize edildi. Son model, 1000 iterasyonluk Bootstrap Resampling ile test edildi.

**Bulgular:**

Eğitim setinde hassasiyet= 0,862, seçicilik= 0,870, doğruluk= 0,863 ve AUC= 0,910 olarak hesaplandı. Benzer şekilde, test setinde bu değerler sırasıyla hassasiyet= 0,848, seçicilik= 0,865, doğruluk= 0,857 ve AUC= 0,907 idi.

**Sonuç:**

Radyomiks parametrelerden oluşturulmuş ve sınırlı bir vaka seti ile eğitilen lojistik regresyon modeli, bilgisayarlı tomografi görüntülerini kullanarak hafif ve ağır şiddetteki amfizem radyolojisini iyi bir seviyede ayırt etti.

## INTRODUCTION

The Global Initiative for Chronic Obstructive Lung Disease (GOLD) criteria used for staging chronic obstructive pulmonary disease (COPD) do not include a radiological parameter ( 1 ). Thoracic CT scans, often used to identify complicating or additional pathologies, do not directly take part in COPD classification, not only due to the avoidance of administering ionizing radiation to the patient but also because of the role of radiological data being reader-dependent and subjective, thus causing significant limitations or biases ( 1 , 2 , 3 ). Radiation doses have significantly reduced over time, with the adoption of low-dose thoracic CT techniques and even ultra-low-dose thoracic CT achieving comparable results ( 4 , 5 ).

To standardize definitions among radiologists, the Fleischner Society classification framework divided centrilobular emphysema, a subtype of COPD, into five radiological subtypes ranging from trace and mild to moderate, confluent, and advanced destructive ( 6 ). A subsequent study reported that individuals with confluent and advanced destructive patterns have a higher risk of mortality during follow-up compared to those with mild and moderate subtypes; however, the study also highlighted the need for comparing visual scoring with quantitative methodologies ( 7 ).

Centrilobular emphysema (CLE) is characterized on thoracic CT scans in the parenchymal window as hypodense areas, resulting from gradual destruction and loss of elastic recoil in the distal small airways and alveoli ( 7 ). The hypodense parenchymal areas described in these studies have been reported to range between -950 and -970 Hounsfield Units (HU) ( 8 ), with some publications reporting values as high as -910 HU ( 9 ). Instead of using a fixed HU value as the evaluation method based on density, it has been suggested to consider the ratio of voxels with lower density than the 15th percentile ( 9 ).

Algorithms for the automatic classification of emphysema patterns have been developed using DL or unsupervised ML methods ( 10 , 11 , 12 , 13 ). In the literature, there is a lack of sufficient information on the effectiveness of lobe-specific volumetric segmentation in assessing the severity of centrilobular emphysema, a disease primarily impacting the upper lobes. Models based on DL and largely “black box” ML algorithms are unable to provide insights into the types of changes in the parenchymal texture that occur as the severity of the emphysema increases at the parenchymal level.

The objective of this study is to investigate the efficacy of a simple and reproducible volumetric segmentation method of the upper lobe segments in determining the severity of centrilobular emphysema on CT scans, while also identifying the changes in radiological texture that arise as emphysema severity increases, using a machine learning algorithm based on logistic regression.

## MATERIALS and METHODS

This retrospective, cross-sectional study was conducted at a single center and received approval from the Institutional Review Board (IRB), with written informed consent waived. The procedures adhered to the ethical guidelines of the 1964 Declaration of Helsinki and its later amendments.

### Study Population

**Figure 1:**
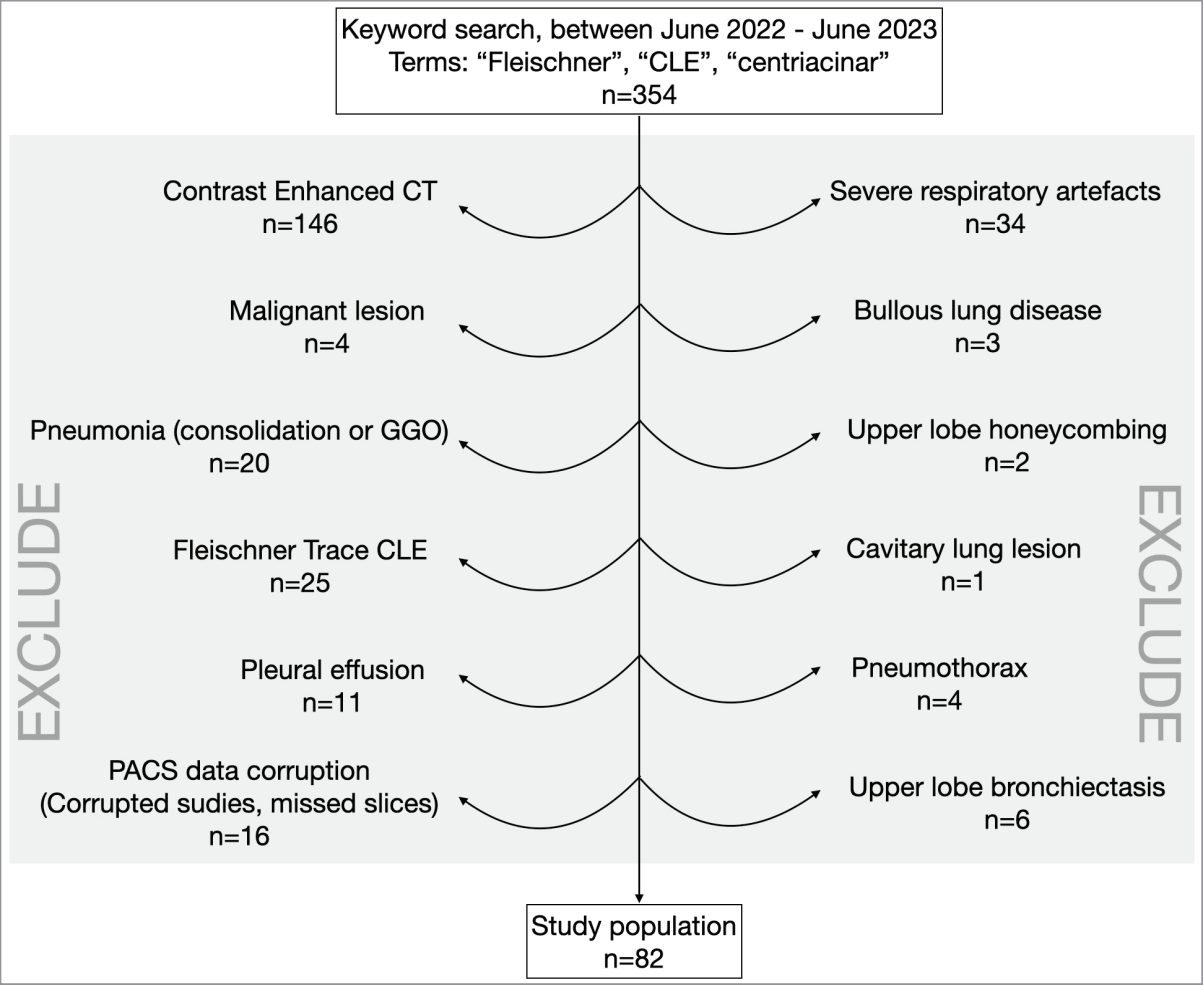
Exclusion criteria

For the study, a total of 354 patients over the age of 18 were selected between June 2022 and June 2023 based on the presence of keywords such as “Fleischner”, “centriacinar”, and “CLE” in their thoracic CT scan reports. The documented emphysema process in the patients’ reports was independently reviewed by the study’s author (MG, with 16 years of experience in thoracic radiology). The most common reasons for exclusion from the study were the administration of contrast medium (n= 146), presence of respiratory artifacts (n= 34), trace CLE lesions according to Fleischner criteria (n= 22), and missing slices in the nonenhanced thoracic CT studies in the PACS database (n= 16). The latter was exclusively observed in patients who had undergone nonenhanced CT scans across multiple anatomic locations due to trauma ( Figure 1 ).

**Figure 2:**
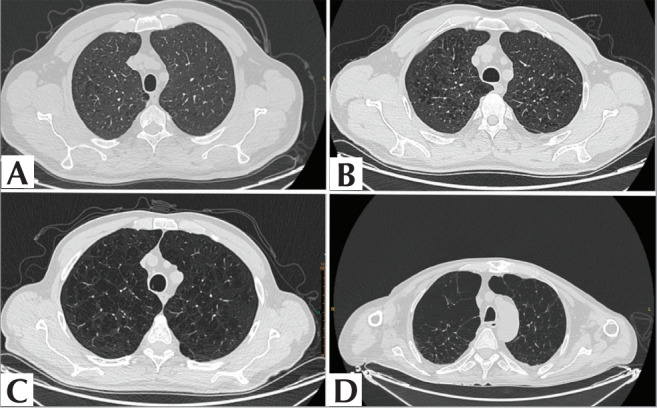
A. Fleischner mild CLE, B. Fleischner moderate CLE, C. Fleischner Confluent CLE, D. Fleischner advanced destructive CLE.

The final study set consisted of 82 patients. Based on the emphysema findings in the thoracic CT scans, the study set was divided into two groups: Group 1, which included cases classified as mild (n= 31) and moderate (n= 14), totaling 45 cases, and Group 2, which included cases classified as confluent (n= 21) and advanced destructive (n= 16), totaling 37 cases, according to the Fleischner emphysema classification ( Figure 2 ).

### CT Protocol

The CT scans were performed from the level of the first rib to the upper renal pole using a 128-detector CT scanner (GE Revolution, GE, Milwaukee, WI). Scans were acquired without contrast, utilizing the following parameters= 100 kV, 110 mAs, a 1.25 mm slice thickness for volumetric study, a 512 x 512 reconstruction matrix, BonePlus kernel, and an adaptive statistical iterative reconstruction (ASIR) of 70%, via body filter.

### Segmentation and Feature Calculation

**Figure 3:**
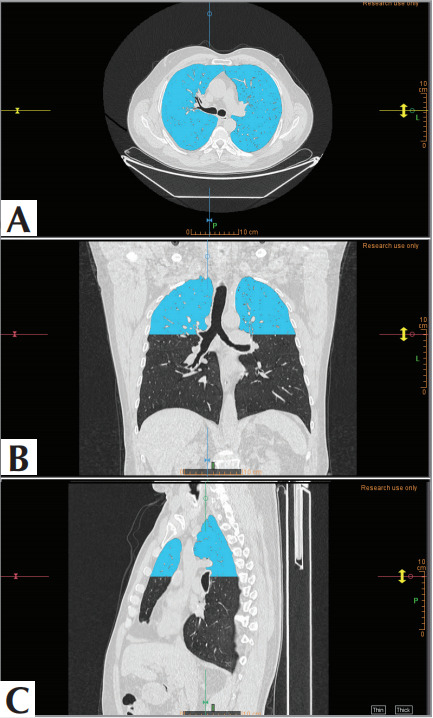
A typical segmentation of the lung in the study. A. Axial view, B. Coronal view, C. Sagittal view.

Both lungs of the patients were volumetrically segmented starting from the apex down to the level of the carina of the trachea. For this purpose, the semi-automatic Region Growing Tool in the Texture Plugin of Olea Sphere 3.0 SP32 (Olea Medical, LaCiotat, FR) software was used. The segmentation could not propagate distal to this level, as areas caudal to the main carina were masked ( Figure 3 ). Since the segmentation achieved had optimized density values for lung parenchyma, the extensions of hilar vascular structures, the chest wall, and mediastinal structures were not sampled; however, the interlobular septa were included in the segmentation. Thus, the segmentation encompassed both apices as well as superior portions of both the upper lobe’s anterior and posterior segments. The reasons for preferring this style of segmentation are as follows: 1) To avoid individual variability in radiomics parameters, which can directly or inversely correlate with the sampling volume by segmenting the entire lung; 2) To ensure that the radiomics findings related computational resources and to keep computation times within reasonable limits.

Following the segmentation, radiomics feature calculations were carried out using the Volume of Interest (VOI) with the Texture Plugin on Olea Sphere 3.0 SP32. In this study, a total of 112 parameters were calculated for each patient, encompassing the domains of original size, original shape, original first order, and second order domains [original gray level co-occurrence matrix (GLCM), original gray level run length matrix (GLRLM), original gray level size zone matrix (GLSZM), original neighboring gray tone difference matrix (NGTDM), original gray level dependence matrix (GLDM)]. Wavelet parameters were not considered.

The following steps were followed for the calculation of the parameters: 1) Since we worked with continuous negative HU values, to prevent the squaring of negative values in the calculations, a voxel array shift of 1024 was added to all voxels. 2) All voxels were resampled to 1 x 1 x 1 mm3 using the B-Spline interpolation method. 3) For grey value discretization, a fixed bin width of 25 was used. 4) In the calculations for second-order matrices, a distance of 1 voxel was chosen, and 13 isotropic displacement vectors with angles of 0, 45, 90, and 135 degrees were employed. 5) Voxel densities to be used for the machine learning algorithm were normalized according to Eq Figure Formul ( 11 ).

where f(x) is the normalized voxel density, x is the original density, μx is the mean density, σx is the standard deviation and S is the scaling factor (set to 1).

### Statistical Analysis

In the study, patients were categorized into two distinct groups for comparative analysis: Group 1, comprising patients with mild emphysema radiological features, and Group 2, comprising patients with severe emphysema radiological features. Both groups were assessed in terms of their mean age, gender distribution, as well as key radiomics parameters pertaining to lesion shape and size. To ascertain the normality of parameter distributions, the Shapiro-Wilk test was utilized. Subsequent group comparisons were conducted using the Student’s t-test for parameters following a normal distribution, and the Mann-Whitney U test for those not conforming to normality. Gender distribution between the groups was statistically evaluated employing Chi-square and Fisher’s exact tests. All statistical analyses were executed using SPSS version 27.0.1.

In this study, only radiomics parameters were used for LR model formation, and clinical parameters were not utilized for creating a hybrid model. Initially, parameter selection was done using the least absolute shrinkage and selection operator (LASSO) regression analysis method. However, due to the small size of the dataset, higher parameter models were further penalized using Bayesian Information Criterion (BIC) and Akaike Information Criterion (AIC). The best logistic regression models with 5, 6, 7, and 8 parameters were then constructed and compared.

The possibility of multicollinearity among the selected model parameters was investigated using the variance inflation factor (VIF) measurement.

The models were separated into train and test sets under Python 3.9 using the sklearn and numPy libraries. The grid search method was employed for hyperparameter optimization. Cross-validation and test metrics were calculated with the specified libraries.

The Hosmer-Lemeshow test was conducted using XLStat 2023 1.6.1410 software (Lumivero, Denver, CO). For the calibration plot, the “rms” library, compiled for the R statistical programming language, was utilized ( 14 ).

For decision curve analysis, we utilized the “dcurves” library, compiled for the R statistical programming language ( 15 ).

**Figure:**
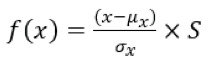


## RESULTS

### Group characteristics

Of the total 82 patients, 45 (36 males, 9 females) were in Group 1, and 37 (33 males, 4 females) were in Group 2. Although males were numerically dominant, no significant difference was observed in terms of gender distribution between the two groups (p= 0.204 Chi-square, p= 0.365 Fisher’s exact test).

The average age of Group 1 was 64.13 ± 13.37, while the average age of Group 2 was 65.86 ± 9.01. No significant difference in age was observed between the two groups (p= 0.466, t-test).

### Comparison of the Shape and Size Features of Segmentations

Upon intergroup comparison, it was observed that the segmentations pertaining to patients in Group 2 demonstrated higher volumes and dimensions, along with lower densities, with these findings being statistically significant ( Table 1 ). The surface area-to-volume ratio of the segmented lung tissue did not exhibit any significant differences between the two groups.

### Logistic Regression Model

The best model selected by the Bayesian Information Criterion (BIC) consisted of five radiomics parameters along with an intercept, making a total of six parameters.

In the model, specific features from both first and second order radiomics domains, the 10th percentile, GLCM information measure of correlation 2, GLSZM size zone non-uniformity normalized, and NGTDM strength, demonstrated statistically significant differences between the two groups ( Table 1 ).

To assess the potential issue of multicollinearity among the model’s parameters, Variance Inflation Factor (VIF) values were calculated utilizing linear regression analysis. All calculated VIF values were found to be below the commonly accepted threshold of 3.0, thereby confirming the absence of multicollinearity concerns within the parameters of the model.

Following the hyperparameter optimization, the optimized form of this model was given in Eq ( 2 ).

Model Prediction= 1 / (1 + exp - (-81.97 – (16.35 × Surface Area-to-Volume Ratio) – (0.12 × 10th percentile) + (15.39 × GLCM Informal Measure of Correlation 2) – (87.73 × GLSZM Size Zone Non-Uniformity Normalized) – (22.01 × NGTDM Strength) ( 2 ).

**Figure 4:**
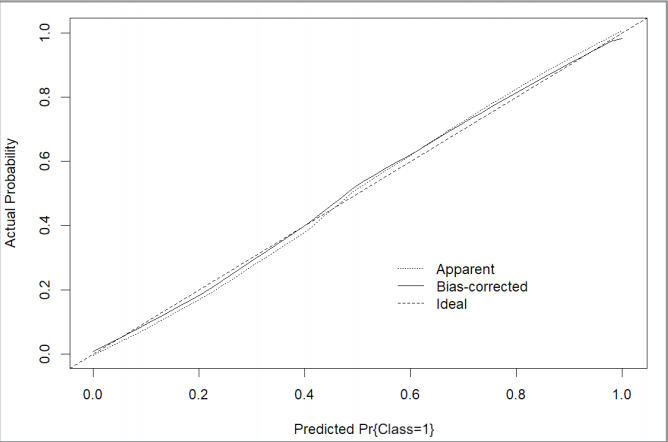
Calibration plot. Apparent and bias-corrected lines are close to the ideal condition. The model is well calibrated.

The Hosmer-Lemeshow test, used to assess the agreement between the predicted risks and the observed outcomes of the model, demonstrated a good fit between the model’s estimated probabilities and the observed results (p= 0.811). Additionally, the calibration plot further corroborated these findings ( Figure 4 ).

The model exhibited high sensitivity, specificity, accuracy, and AUC in both the training and test sets ( Table 2 ). To avoid biased results due to the small size of the case set, the model was subjected to 1000 iterations of bootstrap resampling. The table also provides the 95% confidence intervals (CI) for these metrics.

**Figure 5:**
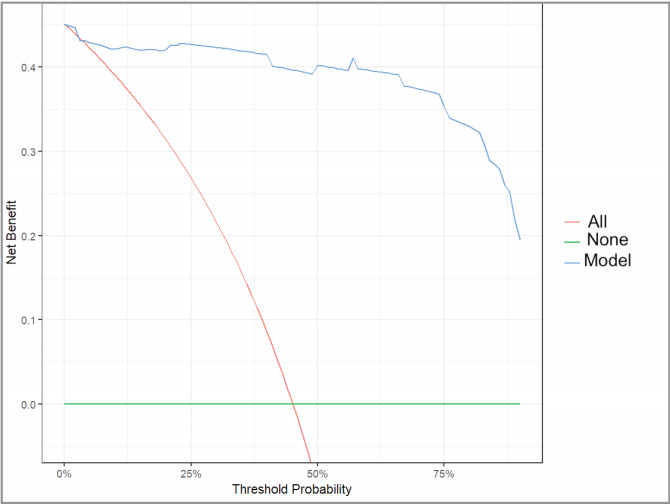
Decision curve analysis. Model (blue) is creating better net benefit under nearly all threshold probabilities. None (green)= Hypothesis predicting there is no severe emphysema in the study group (False negative results and no net benefit). All (red)= Hypothesis predicting all cases in the study group are severe emphysema (False-positive results and limited net benefit).

In the decision curve analysis, which evaluates various threshold probabilities to reflect the trade-off between true positives (benefit) and false positives (harm), the model was found to provide a substantial net benefit across low, medium, and high threshold probability areas ( Figure 5 ).

**Table 1:** Comparison of major shape and size features and model specific parameters between the groups

Feature	Group 1	Group 2	p
Segmentation Volume (mL)	1075 ± 330	1474 ± 442	<0.001^a^
Surface area/Volume ratio	0.146 (0.057)	0.133 (0.062)	0.482^b^
Major axis (mm)	250.74 (25.47)	255.61 (30.78)	0.119^b^
Minimum density (HU)	-1283 (57)	-1328 (42)	<0.001b
10th density (HU)	-970 (32)	-1013 (17)	<0.001b
90th density (HU)	-723 (63)	-787 (75)	<0.001b
Mean density (HU)	-856 (40)	-903 (37)	<0.001b
IMC2	0.370 ± 0.073	0.440 ± 0.083	<0.001^a^
GLSZMNUN	0.446 ± 0.015	0.436 ± 0.015	0.006^a^
Strength	0.780 ± 0.047	0.610 ± 0.023	0.04^a^

Results are provided as mean + SD or median (IQR) according to the distribution of parameters. a: T-test result, b: Mann-Whitney test result. IMC2: GLCM information measure of correlation 2, GLSZMNUN: GLSZM non-uniformity normalized, Strength: NGTDM strength.

**Table 2:** Features of the model in discriminating between mild and severe emphysema

Parameter	p	Odds ratio (95% CI)	Training set*			
			Sensitivity	Specificity	Accuracy	AUC
SA/Vol	0.115	0.383 0.116-1.263	0.862 0.849-0.875	0.870 0.864-0.876	0.863 0.854-0.876	0.910 0.898-0.922
10^th^	0.001	0.014 0.002-0.122				
IMC2	0.007	5.361 1.52-18.278	Test Set*			
GLSZNUN			Sensitivity	Specificity	Accuracy	AUC
Strength	0.027	0.311 0.11-0.877	0.848 0.834-0.682	0.865 0.855-0.875	0.857 0.850-0.865	0.907 0.894-0.919
Intercept	0.088	0.380				

*Results are derived from 1000 iterations of bootstrap resampling and given as mean and 95% CI. SA/Vol: Surface area-to-volume ratio, 10th: 10th percentile, IMC2: GLCM informal measure of correlation 2, GLSZNUN: GLSZM size zone nonuniformity normalized, Strength: NGTDM strength.

## DISCUSSION

In this study, utilizing a dataset with a limited number of cases, we developed a machine-learning model that effectively differentiates between mild and severe emphysema using solely radiological parameters, without the inclusion of any clinical variables. Given that CLE is more commonly observed in smokers and predominantly appears in the upper lung lobes ( 16 ), this model was specifically designed using volumetric segmentation that encompasses a portion of the upper lung lobes.

The relationship between radiomics parameters and the VOI is contingent on the formula used to calculate each parameter. Accordingly, there are parameters that increase with segmentation volume (such as energy), decrease (like coarseness and compactness), or remain constant (e.g., Mean Intensity, Entropy) ( 17 ). Previous studies have reported that the intraclass correlation among radiomics features declines as segmentation volumes increase ( 18 ). Therefore, although “whole lung” segmentations might broaden the sample size, they were not employed due to the anticipated adverse effect on the calculated radiomics parameters.

In a comparative evaluation of parameters related to shape and size domains, it was observed that patients with more severe emphysema exhibited lower HU values in parenchymal density parameters, which aligns with existing literature ( 19 ). Among these parameters, only the 10th percentile was selected by the BIC method for inclusion in the model; however, the interquartile range also appears among the parameters chosen by LASSO. All efforts to reduce the number of parameters were undertaken to protect the model from overfitting. Maintaining a high number of parameters in machine learning models trained on datasets with a low sample size can result in poor performance in test sets due to overfitting ( 20 ). Therefore, it was not possible to use all the parameters selected by LASSO in this study.

In the domain of shape, the surface area-to-volume ratio was a consistent parameter across candidate models that included 6, 7, or 8 radiomics parameters, aside from the selected model. Interestingly, this occurred even though statistically there was no significant difference between the mild and severe emphysema groups for this parameter. This ratio reaches its highest values in objects resembling pyramids or tetrahedra and its lowest in spheres ( 21 ). Within the chest cavity, constrained by the ribcage, it has been demonstrated that lung density and the total volume of emphysematous lesions are linearly related to the surface area-to-volume ratio parameter ( 22 ).

The inclusion of first- and second-order parameters in the model serves to quantify the radiographic representation of emphysematous regions. As the extent of emphysematous areas increases, the relatively heterogeneous parenchyma of normal lung tissue recedes, being supplanted by homogeneous, low-density emphysematous zones. Specifically, parameters such as the 10th percentile, GLCM information measure of correlation 2, GLSZM size zone non-uniformity normalized, and NGTDM Strength all show statistically significant differences between the two groups.

This study has some limitations. First, the study was conducted with a small set of cases. To prevent this from causing biased results, 1000 iterations of bootstrap resampling were used. Second, this study reflects the results of a single center. However, by harmonizing results from other hospitals and other CT scanners ( 23 ), it is possible to eliminate devicerelated footprints and ensure further improvement of the model. Finally, the semi-automatic segmentation tool used in this study requires user intervention, and artificial intelligence algorithms that provide fully automatic segmentation could not be used. In the near future, we aim to develop an automatic lung segmentation algorithm for the institution scanners where the study was carried out.

## CONCLUSION

In conclusion, this study, conducted with a limited dataset, successfully distinguished severe emphysema from mild emphysema in radiological terms. In the future, there is potential to individualize the diagnosis and treatment for COPD patients by incorporating quantified radiological features into clinical data.

## Ethical Committee Approval

This study was approved by the Ankara Bilkent City Hospital Ethical Committee (Decision no: EK-1-23-3973, Date: 06.09.2023).

## CONFLICT of INTEREST

The authors declare that they have no conflict of interest.

## AUTHORSHIP CONTRIBUTIONS

Concept/Design: MG

Analysis/Interpretation: MG

Data acqusition: MG

Writing: MG

Clinical Revision: MG

Final Approval: MG
